# Supplementary material for: Smooth Interpolating Curves with Local Control and Monotone Alternating Curvature
Source: Comput Graph Forum. 2022 Oct 6;41(5):25–38. doi: 10.1111/cgf.14600 (PMC9827861; doi:10.1111/cgf.14600)
Supplement: Supplementary file 1 — Supplement Material [file CGF-41-25-s001.zip › Local-Smooth-Interpolating-MonoCurvature/extern/clothoids/docs/api-matlab/program_listing_file_BiarcList.m.html]

Program Listing for File BiarcList.m — Clothoids v2.0.9

### Navigation

- index
- toc
- Clothoids »
- Program Listing for File BiarcList.m

# Program Listing for File BiarcList.m¶

↰ Return to documentation for file (`BiarcList.m`)

```
classdef BiarcList < CurveBase

  methods
    % - - - - - - - - - - - - - - - - - - - - - - - - - - - - - - - - - - -
    %> Create a new C++ class instance for the list of biarc object
    %>
    %> **Usage:**
    %>
    %> \rst
    %> .. code-block:: matlab
    %>
    %>   self = BiarcList();
    %>
    %> \endrst
    %>
    %> **On output:**
    %>
    %> - self: reference handle to the object instance
    %>
    function self = BiarcList()
      self@CurveBase( 'BiarcListMexWrapper' );
      self.objectHandle = BiarcListMexWrapper( 'new' );
    end
    % - - - - - - - - - - - - - - - - - - - - - - - - - - - - - - - - - - -
    %> Return the string `"BiarcList"`with the name of the object class
    %>
    function str = is_type( ~ )
      str = 'BiarcList';
    end
    % - - - - - - - - - - - - - - - - - - - - - - - - - - - - - - - - - - -
    %> Reserve memory for `N` biarc
    %>
    %> **Usage:**
    %>
    %> \rst
    %> .. code-block:: matlab
    %>
    %>   ref.reserve(N);
    %>
    %> \endrst
    %>
    %>
    function reserve( self, N )
      BiarcListMexWrapper( 'reserve', self.objectHandle, N );
    end
    % - - - - - - - - - - - - - - - - - - - - - - - - - - - - - - - - - - -
    %> Append to the BiarcList another biarc.
    %> The biarc is obtained setting the final postion and angle while
    %> the initial position and angle are taken from the last biarc on
    %> the biarc list.
    %> Another possibility is to push a biarc is obtained
    %> by passing initial and final positions, initial and final angles.
    %>
    %> **Usage:**
    %>
    %> \rst
    %> .. code-block:: matlab
    %>
    %>   ref.push_back(x1,y1,theta1);
    %>   ref.push_back(x0,y0,theta0,x1,y1,theta1);
    %> \endrst
    %>
    %> - `x0`, `y0`: initial position of the biarc to be appended
    %> - `theta0`  : initial angle of the biarc to be appended
    %> - `x1`, `y1`: final position of the biarc to be appended
    %> - `theta1`  : final angle of the biarc to be appended
    %>
    function push_back( self, varargin )
      BiarcListMexWrapper( 'push_back_G1', self.objectHandle, varargin{:} );
    end
    % - - - - - - - - - - - - - - - - - - - - - - - - - - - - - - - - - - -
    %> Get the biarc at position `k`.
    %> The biarc is returned as a biarc object or the data
    %> defining the biarc.
    %>
    %> \rst
    %> .. code-block:: matlab
    %>
    %>   BA = ref.get(k); % get the biarc object
    %>   [ x0, y0, theta0, x1, y1, theta1 ] = ref.get(k); % get the biarc data
    %> \endrst
    %>
    function varargout = get( self, k )
      [ x0, y0, theta0, x1, y1, theta1 ] = BiarcListMexWrapper( 'get', self.objectHandle, k );
      if nargout == 1
        varargout{1} = Biarc( x0, y0, theta0, x1, y1, theta1 );
      elseif nargout == 6
        varargout{1} = x0;
        varargout{2} = y0;
        varargout{3} = theta0;
        varargout{4} = x1;
        varargout{5} = y1;
        varargout{6} = theta1;
      else
        error('expected 1 or 6 outout arguments');
      end
    end
    % - - - - - - - - - - - - - - - - - - - - - - - - - - - - - - - - - - -
    %> Append a biarc or a biarc list.
    %>
    %> \rst
    %> .. code-block:: matlab
    %>
    %>   ba = Biarc( ... );
    %>   % ....
    %>   ref.append(ba); % append biarc
    %>
    %>   blist = BiarcList();
    %>   % ...
    %>   ref.append(blist); % append biarc list
    %> \endrst
    %>
    function append( self, lst )
      if lst.is_type() == 'BiarcList'
        for k=1:lst.numSegments()
          [ x0, y0, theta0, x1, y1, theta1 ] = lst.get(k);
          self.push_back( x0, y0, theta0, x1, y1, theta1 );
        end
      elseif lst.is_type() == 'BiArc'
        [ x0, y0, theta0, x1, y1, theta1 ] = lst.getData();
        self.push_back( x0, y0, theta0, x1, y1, theta1 );
      else
        error('expected a biarc or a biarc list as input argument');
      end
    end
    % - - - - - - - - - - - - - - - - - - - - - - - - - - - - - - - - - - -
    %> Return the list of points (initial and final) of the biarcs
    %>
    %> \rst
    %> .. code-block:: matlab
    %>
    %>   [ x, y ] = ref.getXY();
    %> \endrst
    %>
    function [ x, y ] = getXY( self )
      [ x, y ] = BiarcListMexWrapper( 'getXY', self.objectHandle );
    end
    % - - - - - - - - - - - - - - - - - - - - - - - - - - - - - - - - - - -
    %> Return number of biarc in the list
    %>
    %> \rst
    %> .. code-block:: matlab
    %>
    %>   nseg = ref.numSegments();
    %> \endrst
    %>
    function N = numSegments( self )
      N = BiarcListMexWrapper( 'numSegments', self.objectHandle );
    end
    % - - - - - - - - - - - - - - - - - - - - - - - - - - - - - - - - - - -
    %> Build a biarc list given a set of points and if available
    %> the angles at the points. If the angles are missing the angle
    %> at a node is computed by building the circle passing by 3
    %> consecutive points. The node is the middle point.
    %>
    %> \rst
    %> .. code-block:: matlab
    %>
    %>   ref.build_G1(x,y);
    %>   ref.build_G1(x,y,theta);
    %> \endrst
    %>
    %> - `x`, `y`: vectors of `x` and `y` coordinates of the nodes
    %> - `thetas`: angles at the nodes
    %>
    function ok = build_G1( self, varargin )
      ok = BiarcListMexWrapper( 'build_G1', self.objectHandle, varargin{:} );
    end
    % - - - - - - - - - - - - - - - - - - - - - - - - - - - - - - - - - - -
    %> Build the angles a the list of nodes.
    %> The angle at a node is computed by building the circle passing by 3
    %> consecutive points. The node is the middle point.
    %>
    %> \rst
    %> .. code-block:: matlab
    %>
    %>   thetas = ref.build_theta(x,y);
    %> \endrst
    %>
    %> - `x`, `y`: vectors of `x` and `y` coordinates of the nodes
    %>
    function [theta,ok] = build_theta( self, x, y )
      [theta,ok] = ...
        BiarcListMexWrapper( 'build_theta', self.objectHandle, x, y );
    end
    % - - - - - - - - - - - - - - - - - - - - - - - - - - - - - - - - - - -
    function info( self )
      BiarcListMexWrapper( 'info', self.objectHandle );
    end
    % - - - - - - - - - - - - - - - - - - - - - - - - - - - - - - - - - - -
    %> Find curvilinear coordinates of inputs points
    %>
    %> \rst
    %> .. code-block:: matlab
    %>
    %>   [ s, t, ipos ] = ref.find_coord1(x,y);
    %> \endrst
    %>
    %> **Input:**
    %>
    %> - `x`, `y`: vectors of `x` and `y` coordinates of the poinst
    %>
    %> **Output:**
    %>
    %> - `s`, `t` : curvilinar coordinates of the points
    %> - `ipos`   : the segment with point at minimal distance, otherwise -(idx+1)
    %>   if (x,y) cannot be projected orthogonally on the segment
    %>
    function [ s, t, ipos ] = find_coord1( self, x, y )
      [ s, t, ipos ] = BiarcListMexWrapper( 'findST1', self.objectHandle, x, y );
    end
    % - - - - - - - - - - - - - - - - - - - - - - - - - - - - - - - - - - -
    %> Plot the biarc list
    %>
    %> **Usage:**
    %>
    %> \rst
    %> .. code-block:: matlab
    %>
    %>   ref.plot();
    %>   ref.plot( npts );
    %>
    %>   fmt1 = {'Color','blue','Linewidth',2}; % first arc of the biarc
    %>   fmt2 = {'Color','red','Linewidth',2};  % second arc of the biarc
    %>   ref.plot( npts, fmt1, fmt2 );
    %>
    %> \endrst
    %>
    %> - `npts`: number of sampling points for plotting
    %> - `fmt1`: format of the first arc
    %> - `fmt2`: format of the second arc
    %>
    function plot( self, varargin )
      if nargin > 1
        npts = varargin{1};
      else
        npts = 400;
      end
      if nargin > 2
        fmt1 = varargin{2};
      else
        fmt1 = {'Color','red','LineWidth',3};
      end
      if nargin > 3
        fmt2 = varargin{3};
      else
        fmt2 = {'Color','blue','LineWidth',3};
      end
      for k=1:1:self.numSegments()
        C = self.get(k);
        C.plot( npts, fmt1, fmt2 );
        hold on;
      end
    end
    % - - - - - - - - - - - - - - - - - - - - - - - - - - - - - - - - - - -
    %> Plot the biarc list with offset
    %>
    %> **Usage:**
    %>
    %> \rst
    %> .. code-block:: matlab
    %>
    %>   ref.plot_offs( offs, npts );
    %>
    %>   fmt1 = {'Color','blue','Linewidth',2}; % first arc of the biarc
    %>   fmt2 = {'Color','red','Linewidth',2};  % second arc of the biarc
    %>   ref.plot_offs( offs, npts, fmt1, fmt2 );
    %>
    %> \endrst
    %>
    %> - `npts`: number of sampling points for plotting
    %> - `fmt1`: format of the first arc
    %> - `fmt2`: format of the second arc
    %> - `offs`: offset used in the plotting
    %>
    function plot_offs( self, offs, npts, varargin )
      if nargin > 3
        fmt1 = varargin{1};
      else
        fmt1 = {'Color','red','LineWidth',3};
      end
      if nargin > 4
        fmt2 = varargin{2};
      else
        fmt2 = {'Color','blue','LineWidth',3};
      end
      for k=1:self.numSegments()
        C = self.get(k);
        C.plot_offs( offs, npts, fmt1, fmt2 );
        hold on;
      end
    end
    % - - - - - - - - - - - - - - - - - - - - - - - - - - - - - - - - - - -
    %> Plot the curvature of the biarc list
    %>
    %> **Usage:**
    %>
    %> \rst
    %> .. code-block:: matlab
    %>
    %>   ref.plotCurvature( npts );
    %>
    %>   fmt1 = {'Color','blue','Linewidth',2};
    %>   fmt2 = {'Color','red','Linewidth',2};
    %>   ref.plotCurvature( npts, fmt1, fmt2 );
    %>
    %> \endrst
    %>
    %> - `npts`: number of sampling points for plotting
    %> - `fmt1`: format of the first arc
    %> - `fmt2`: format of the second arc
    %>
    function plotCurvature( self, npts, varargin )
      if nargin > 2
        fmt1 = varargin{1};
      else
        fmt1 = {'Color','red','LineWidth',3};
      end
      if nargin > 3
        fmt2 = varargin{2};
      else
        fmt2 = {'Color','blue','LineWidth',3};
      end
      s0 = 0;
      for k=1:self.numSegments()
        C  = self.get(k);
        ss = 0:C.length()/npts:C.length();
        [~,~,~,kappa] = C.evaluate(ss);
        if mod(k,2) == 0
          plot( s0+ss, kappa, fmt1{:} );
        else
          plot( s0+ss, kappa, fmt2{:} );
        end
        s0 = s0+ss(end);
        hold on;
      end
    end
    % - - - - - - - - - - - - - - - - - - - - - - - - - - - - - - - - - - -
    %> Plot the angle of the biarc list
    %>
    %> **Usage:**
    %>
    %> \rst
    %> .. code-block:: matlab
    %>
    %>   ref.plotAngle( npts );
    %>
    %>   fmt1 = {'Color','blue','Linewidth',2};
    %>   fmt2 = {'Color','red','Linewidth',2};
    %>   ref.plotAngle( npts, fmt1, fmt2 );
    %>
    %> \endrst
    %>
    %> - `npts`: number of sampling points for plotting
    %> - `fmt1`: format of the first arc
    %> - `fmt2`: format of the second arc
    %>
    function plotAngle( self, npts, varargin )
      if nargin > 2
        fmt1 = varargin{1};
      else
        fmt1 = {'Color','red','LineWidth',3};
      end
      if nargin > 3
        fmt2 = varargin{2};
      else
        fmt2 = {'Color','blue','LineWidth',3};
      end
      s0 = 0;
      for k=1:self.numSegments()
        C  = self.get(k);
        ss = 0:C.length()/npts:C.length();
        [~,~,theta,~] = C.evaluate(ss);
        if mod(k,2) == 0
          plot( s0+ss, theta, fmt1{:} );
        else
          plot( s0+ss, theta, fmt2{:} );
        end
        s0 = s0+ss(end);
        hold on;
      end
    end
    % - - - - - - - - - - - - - - - - - - - - - - - - - - - - - - - - - - -
    %> Plot the normal of the biarc list
    %>
    %> **Usage:**
    %>
    %> \rst
    %> .. code-block:: matlab
    %>
    %>   ref.plotNormal( step, len );
    %>
    %> \endrst
    %>
    %> - `step`: number of sampling normals
    %> - `len`:  length of the plotted normal
    %>
    function plotNormal( self, step, len )
      for k=1:self.numSegments()
        C = self.get(k);
        C.plotNormal( step, len );
      end
    end
    % - - - - - - - - - - - - - - - - - - - - - - - - - - - - - - - - - - -
    %> Save the biarc list sampled on a file
    %>
    %> **Usage:**
    %>
    %> \rst
    %> .. code-block:: matlab
    %>
    %>   ref.save( filename, ds );
    %>
    %> \endrst
    %>
    %> - `filename`: file name
    %> - `ds`:       sample point every `ds`
    %>
    %> the file is of the form
    %> \rst
    %> .. code-block:: text
    %>
    %>   X Y THETA
    %>   0 0 1.2
    %>   ...
    %>   ...
    %>
    %> \endrst
    %>
    function save( self, filename, ds )
      fd = fopen( filename, 'w' );
      L  = self.length();
      n  = ceil( L / ds );
      fprintf(fd,'X\tY\tTHETA\n');
      for k=1:n
        s = (k-1)*L/(n-1);
        [x,y,theta] = self.evaluate( s );
        fprintf(fd,'%20.10g\t%20.10g\t%20.10g\n',x,y,theta);
      end
      fclose(fd);
    end
  end
end
```

### Quick search

### Table of Contents

- Matlab Interface Manual
- C++ API
- MATLAB API

«
hide menu

menu
sidebar
»

### Navigation

- index
- toc
- Clothoids »
- Program Listing for File BiarcList.m

© Copyright 2021, Enrico Bertolazzi and Marco Frego.
Created using Sphinx 4.2.0.
